# Hyperprolactinemia Secondary to Allergic Fungal Sinusitis Compressing the Pituitary Gland

**DOI:** 10.1155/2016/7260707

**Published:** 2016-02-21

**Authors:** Nikita Chapurin, Cynthia Wang, David M. Steinberg, David W. Jang

**Affiliations:** ^1^Division of Head and Neck Surgery & Communication Sciences, Department of Surgery, Duke University Medical Center, Durham, NC 27710, USA; ^2^Department of Pathology, Duke University Medical Center, Durham, NC 27710, USA

## Abstract

*Objective*. We aim to describe the first case in the literature of allergic fungal sinusitis (AFS) presenting with hyperprolactinemia due to compression of the pituitary gland.* Case Presentation*. A 37-year-old female presented with bilateral galactorrhea and occipital headaches of several weeks. Workup revealed elevated prolactin of 94.4, negative pregnancy test, and normal thyroid function. MRI and CT demonstrated a 5.0 × 2.7 × 2.5 cm heterogeneous expansile mass in the right sphenoid sinus with no pituitary adenoma as originally suspected. Patient was placed on cabergoline for symptomatic control until definitive treatment.* Results*. The patient underwent right endoscopic sphenoidotomy, which revealed nasal polyps and fungal debris in the sphenoid sinus, consistent with AFS. There was bony erosion of the sella and clivus. Pathology and microbiology were consistent with allergic fungal sinusitis caused by* Curvularia* species. Prolactin levels normalized four weeks after surgery with resolution of symptoms.* Conclusion*. Functional endoscopic sinus surgery alone was able to reverse the patient's pituitary dysfunction. To our knowledge, this is the first case of AFS presenting as hyperprolactinemia due to pituitary compression.

## 1. Introduction

Causes of pathologic hyperprolactinemia may include pituitary prolactinomas, induction by medication, and numerous endocrine disorders [1, 2]. Here we aim to describe the first known case in the literature of allergic fungal sinusitis (AFS) presenting with hyperprolactinemia due to compression of the pituitary gland. It describes a unique presentation of an otherwise common rhinologic disease.

AFS is a relatively new and incompletely understood clinical entity and is therefore frequently misdiagnosed. It is a form of fungal sinusitis characterized by presence of polyposis, fungal debris, and Type 1 hypersensitivity [3]. It is a distinct clinical entity from invasive fungal disease, because there is no histologic or clinical evidence of tissue invasion [4]. AFS has an insidious onset, often without the usual sinonasal symptoms. One of the hallmark features of AFS is unilateral involvement, and extensive bone erosion of the skull base and orbit [5]. While vision, proptosis, and mental status changes have been described as possible presentations of the disease, we aim to present the first case of a neuroendocrine disorder caused by AFS secondary to compression of the pituitary gland. By presenting this case we hope to emphasize the sequelae of extensive progression of AFS and to illustrate an unusual presentation of an already frequently misdiagnosed clinical entity.

## 2. Case Presentation

**Figure 1:**
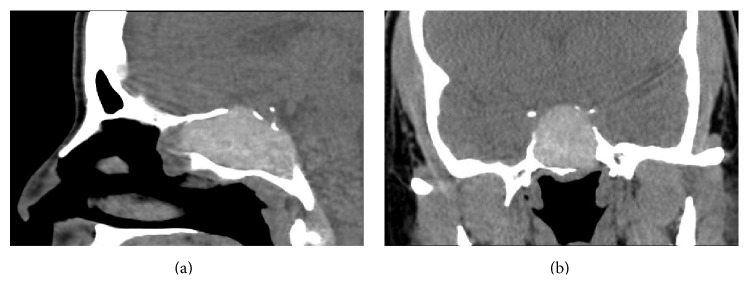
Sinus CT with sagittal (a) and coronal (b) reconstructions demonstrating a 5.0 × 2.7 × 2.5 cm hyperdense sphenoid mass with marked sphenoid expansion, suggestive of allergic fungal sinusitis. There is erosion of the sella, clivus, and a portion of the planum sphenoidale.

**Figure 2:**
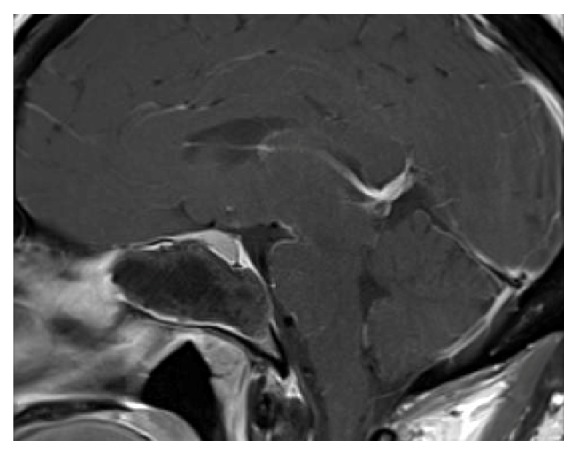
MRI of brain with sagittal view demonstrating expansile lesion centered in sphenoid sinus. The area with the signal void represents fungal debris. There is noticeable mass effect upon the sella with superior deviation of the pituitary gland and kinking of the infundibulum.

A 37-year-old female with a history of type 2 diabetes presented with bilateral galactorrhea and occipital headaches of several weeks. Initial workup revealed elevated prolactin of 94.4 (normal range: 3.3–26.8 ng/mL), negative pregnancy test, and normal thyroid function. The patient did not have a history of renal or liver disease and was not on a medication that would contribute to hyperprolactinemia. An endocrine workup was performed and demonstrated normal levels of other hormones (ACTH: 12 pg/mL, TSH: 0.34 *μ*IU/mL, free T4: 0.66 ng/dL, IGF-1: 113 ng/mL, and cortisol: 6.1 *μ*g/dL). Patient's endocrinologist initiated cabergoline 0.25 mg twice a week for prolactin suppression and consulted neurosurgery for a probable prolactinoma. CT scan demonstrated a 5.0 × 2.7 × 2.5 cm hyperdense expansile mass in the right sphenoid sinus with erosion of the sella and clivus (Figure 1). An MRI with contrast demonstrated a large signal void in the sphenoid sinus. There was mass effect upon the sella with superior deviation of the pituitary gland and kinking of the infundibulum (Figure 2).

**Figure 3:**
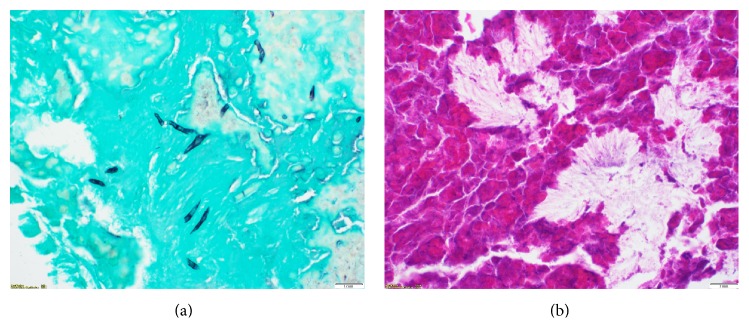
(a) GMS stain demonstrating scattered noninvasive fungal hyphae. (b) H&E stain showing allergic mucin and lamellated inflammatory debris with numerous eosinophils and Charcot-Leyden crystals.

The patient was then referred to an otolaryngologist. Nasal endoscopy revealed polyps in the right sphenoethmoid recess. The patient subsequently underwent right endoscopic sphenoidotomy with complete removal of polyps and fungal debris. There was extensive bony erosion of the sella and clivus without evidence of mucosal invasion. Pathology demonstrated presence of allergic mucin, numerous eosinophils, Charcot-Leyden crystals, and noninvasive fungal hyphae visible on GMS stains (Figure 3). Fungal culture confirmed the presence of* Curvularia* species. These findings and imaging were all consistent with diagnosis of allergic fungal sinusitis. Prolactin levels normalized within 4 weeks after surgery, and the patient had an uneventful recovery with resolution of her symptoms at 1 year of follow-up. Postoperatively, the patient was managed medically with twice-daily high-volume budesonide irrigations. No follow-up imaging was obtained since the patient became asymptomatic and office nasal endoscopy demonstrated appropriate recovery.

## 3. Discussion

AFS is a form of noninvasive fungal disease that is a relatively newly recognized and incompletely understood disease entity. High index of suspicion is critical in early clinical diagnosis since this is an insidious disease [3]. Major criteria for diagnosis established by Bent and Kuhn include presence of nasal polyposis, characteristic sinus CT findings of heterogeneous hyperdensities that are often unilateral and asymmetric, Type 1 hypersensitivity, eosinophilic mucin, and positive fungal stain or culture [6]. The pathophysiology of AFS is believed to involve a Type 1 hypersensitivity reaction against common colonizing fungus [7]. While the most common presenting symptoms of AFS include nasal obstruction and anosmia, up to 53% of patients may present with more progressive disease, characterized by bony erosion and remodeling of the skull base [5]. In the case of the described patient, bony erosion involved the sella and clivus (Figure 1), with subsequent deviation of the pituitary gland and kinking of the infundibulum (Figure 2). Although AFS is typically responsive to systemic steroids, surgical management (i.e., functional endoscopic sinus surgery) plays an important role. Postoperatively, topical steroids and immunotherapy are important, but antifungal agents are not typically used [8, 9].

Synthesis and secretion of prolactin are suppressed by the hypothalamic dopamine traversing the portal venous system that acts upon the lactotroph D2 receptors. In contrast, hormones such as thyrotropin-releasing hormone (TRH), epidermal growth factor, and dopamine receptor antagonists induce prolactin secretion [10]. In this patient, the mass effect on the infundibulum was responsible for blocking the inhibitory tuberoinfundibular dopamine pathway, resulting in uninhibited pituitary prolactin release and manifesting as galactorrhea. Although the patient was started on cabergoline for management of galactorrhea prior to the diagnosis of allergic fungal sinusitis, surgical removal of fungal material was critical in normalizing prolactin levels.

In conclusion, we report the first known case of hyperprolactinemia due to AFS of the sphenoid sinus, with endocrine dysregulation resulting from compression of the pituitary gland. AFS was diagnosed both radiographically and histologically, with endoscopic sinus surgery providing definitive treatment.
